# Supplementary material for: The Humanization and Maturation of an Anti-PrPc Antibody
Source: Bioengineering (Basel). 2024 Feb 29;11(3):242. doi: 10.3390/bioengineering11030242 (PMC10968383; doi:10.3390/bioengineering11030242)
Supplement: Supplementary file 1 [file bioengineering-11-00242-s001.zip › bioengineering-2832572-supplementary.pdf]

bioengineering

Supplementary Materials

The Humanization and Maturation of an Anti-PrPc Antibody

Cheng Zhang [1,2,†], Fanlei Ran [1,2,†], Lei Du [3,4,†], Xiaohui Wang [3], Lei Liu [3,4], Jinming Liu [5], Quan Chen [5], Yang Cao [6], Lijun Bi [1,2,*] and Haiying Hang [1,2,*]

[1] Key Laboratory of RNA Biology, Institute of Biophysics, Chinese Academy of Sciences, Beijing 100101, China; zhangc1mark@163.com (C.Z.); ranfanlei88@163.com (F.R.)
[2] University of Chinese Academy of Sciences, Beijing 100049, China
[3] The State Key Laboratory of Membrane Biology, Institute of Zoology, Chinese Academy of Sciences, Beijing 100101, China; dul@ioz.ac.cn (L.D.); wangxhui@ioz.ac.cn (X.W.); liulei@ioz.ac.cn (L.L.)
[4] Beijing Institute for Stem Cell and Regenerative Medicine, Beijing 100101, China
[5] The State Key Laboratory of Medicinal Chemical Biology, College of Life Sciences, Nankai University, Tianjin 300071, China; 18630864841@163.com (J.L.); chenq@nankai.edu.cn (Q.C.)
[6] Center of Growth, Metabolism and Aging, Key Laboratory of Bio-Resources and Eco-Environment of Ministry of Education, College of Life Sciences, Sichuan University, Chengdu 610064, China; cao@scu.edu.cn
[*] Correspondence: blj@ibp.ac.cn (L.B.); hhang91@163.com (H.H.)
[†] These authors contributed equally to this work.

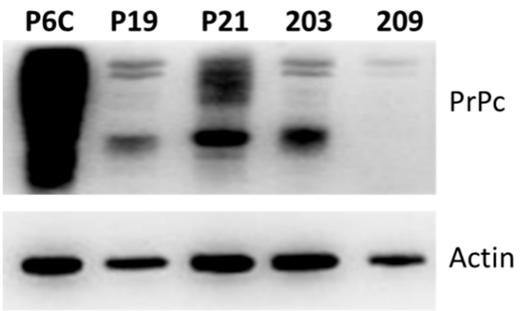

Figure S1. Western blotting of P6C, P6C-KO-P19, P6C-KO-P21 (which is not used in the article), P6C-KO-203, P6C-KO-209 by Clone 6.

A

```
Clone 6:   EVQLQQSGPELVKPGASVKMSCKASGYTFTDYYMKWVKQSHGKSLEWIGDINPNNGDTFYNQ
IGH:       EVQLVQSGAEVKKPGATVKISCKVSGYTFTDYYMHWVQQAPGKGLEWMGLVDPEDGETIYAE
HAb 6:     EVQLVQSGAEVKKPGATVKMSCKASGYTFTDYYMKWVKQAPGKSLEWIGDINPNNGDTFYNQ
HAb 6b:    EVQLVQSGAEVKKPGATVKMSCKASGYTFTDYYMKWVKQAPGKGLEWIGDINPNNGDTFYNE
HAb 6c:    EVQLVQSGAEVKKPGATVKISCKASGYTFTDYYMKWVQQAPGKGLEWMGLVNPNNGDTFYNE

Clone 6:   KFKGKATLTVDKSSNTAYMQLNSLTSEDSAVYYCAKPG----RTYWGQGTLVTVS
IGH:       KFQGRVTITADTSTDTAYMELSSLRSEDTAVYYCATGGIAVAAPYWGQGTLVTVS
HAb 6:     KFQGRATLTVDKSSNTAYMELSSLRSEDTAVYYCAKPG----RTYWGQGTLVTVS
HAb 6b:    KFQGRATLTVDKSTNTAYMELSSLRSEDTAVYYCAKPG----RTYWGQGTLVTVS
HAb 6c:    KFQGRVTITVDKSTNTAYMELSSLRSEDTAVYYCATPG----RTYWGQGTLVTVS
```

B

```
Clone 6:   DIVLTQSPSSLSVSAGEKVTMSCKSSQSLLDSGNQKNYLVWYQQKPGQPPKLLIYGASTRES
IGL:       DIVMTQSPDSLAVSLGERATINCKSSQSVLYSSNNKNYLAWYQQKPGQPPKLLIYWASTRES
HAb 6:     DIVLTQSPDSLAVSLGERVTMSCKSSQSLLDSGNQKNYLVWYQQKPGQPPKLLIYGASTRES
HAb 6b:    DIVLTQSPDSLAVSLGERVTMSCKSSQSLLDSGNQKNYLVWYQQKPGQPPKLLIYGASTRES
HAb 6c:    DIVLTQSPDSLAVSLGERATISCKSSQSLLDSGNQKNYLVWYQQKPGQPPKLLIYGASTRES

Clone 6:   GVPDRFTGSGSGTDFTLTISSVQPEDLAVYYCQNDHSYP-LTFGAGTKLELKR
IGL:       GVPDRFSGSGSGTDFTLTISSLQAEDVAVYYCQQYYSTPSLTFAGGTKVEIK-
HAb 6:     GVPDRFTGSGSGTDFTLTISSVQAEDVAVYYCQNDHSYP-LTFGAGTKLELKR
HAb 6b:    GVPDRFSGSGSGTDFTLTISSVQAEDVAVYYCQNDHSYP-LTFGAGTKLELKR
HAb 6c:    GVPDRFSGSGSGTDFTLTISSLQAEDVAVYYCQNDHSYP-LTFGAGTKLELKR
```

Figure S2. Humanization of mouse anti-PrPc antibody Clone 6. (**A**) Sequences of antibody heavy chain variable fragments. The IGH is the most similar human germline sequence with Clone 6. (**B**) Sequences of antibody light chain variable fragments (IGL). The IGL is the most similar human germline sequence with Clone 6. Red letters representing the amino acid residues of the human antibodies different from those of Clone 6. - stands for no corresponding amino acids.

Table S1. The sequence of primers.

primers	sequence
PrPc NHE1 F	GTACATGCTAGCGCCACCATGGCGAACCTTGGCTGCTGG
PrPc XHO1 R	GTACATCTCGAGCCATCATCCCACTATCAGGAAGATGAGG
PrPc Fc F1	GTACATAAGCTTGCCACCATGGCGAACCTTGGC
PrPc Fc R2	GTACATCTCGAGTTATCACTTGCCTGGGCTCAGGG
PrPc Fc R1	GTCACAGGACTTAGGGGGCTCTCCTCTCTGGTAATAGGCCTG
PrPc Fc F2	CAGGCCTATTACCAGAGAGGAGAGCCCCCTAAGTCCTGTGAC
PrPc hu heavy A16D F	GAAGAAGCCAGGCGATACCGTGAAGATG
PrPc hu heavy A16D R	CATCTTCACGGTATCGCCTGGCTTCTTC
A97V F	GTGTACTATTGTGTTAAGCCAGGAAGGAC
A97V R	GTCCTTCCTGGCTTAACACAATAGTACAC
41insert4AA F	GGGTGAAGCAGGCTCCCGGCAAGGCTCCCGGCAAGTCCCTGGAGTGG
41insert4AA R	CCACTCCAGGGACTTGCCGGGAGCCTTGCCGGGAGCCTGCTTCACCC
Hind3-kozak-SP-F	AATATAAGCTTGCCACCATGACCCGGCTGACCGTGCTG
genome R	CTGCGTGTCCTGGCCCACAGC
gRNA	CGAGACCGACGTTAAGATGA
